# A Method of Pulp Canal Filling

**Published:** 1899-10

**Authors:** G. W. Knight

**Affiliations:** Brooklyn.


ARTICLE V.

### A METHOD OF PULP CANAL FILLING.

G. W. KNIGHT, D. D. S., BROOKYN.

The subject of dead teeth from the standpoint of discussion is as old as the hills, and an apology is instinctively suggested when the matter is even mentioned. Yet it is not as threadbare as might be supposed after passing through so many hands; the question is vital to all, being as it is an every-day problem demanding an accurate solution, and I have faith to believe that there are many whom it will interest.

The history of the literature and discussion of this topic is entertaining as well as instructive, and leads to two suspicions. First, that there is more than one royal road to success; and, secondly, that many roads to failure are made so by the manner in which they are traveled. It is safe to assert that, all positive statements to the contrary notwithstanding, there are ways and materials which can be employed to reach the desired end, and not one way and one material only. It is an equally safe statement that any method and any material may become a means to disaster if employed unskilfully.

It is not the purpose of this paper to touch on the opening of the root canal; it presumes that all that has been properly attended to. Schreier method and Donaldson cleansers, if you will, but the greatest care and patience surely, and the canal cleaned, sterilized and opened to the very best of the operator's ability.

Nor is it intended to discuss the relative merits of materials which might be employed for the purpose of filling the canal. The aim is simply to present a method by which the operator can feel, when he has finished the work, that the canal is filled and filled as it should be.

There are certain qualities which the ideal canal filling should possess, and the first of these is adaptability. Time, care and labor are spent in opening the canal and getting the root into a healthy condition; it is discouraging to employ a material in the filling process which possesses the possibility of clogging the canal when half way to the apex, thereby leaving the remaining and troublesome end open to invite a return of putrefactive conditions and their resultant evils. The material should also adhere closely to the walls of the canal, sealing every crevice and as far as possible the microscopic openings of the canaliculi, thereby acting as a mechanical preventive of putrefaction by excluding the ingress and egress of air or gases to or from these tiny channels.

Secondly, compatibility. When the apical foramen is large, or when extra effort has been made to force the filling home, a slight portion of the filling may occasionally find its way through the opening into the tissue beyond. Any material would, under these conditions, act as a foreign body and be treated as such, causing inflammation. It is desirable, therefore, to use a substance which will be absorbed or become readily acceptable under such circumstances. If the substance has antiseptic properties it will be additionally valuable, continuing the sterilization of the canal if that important work has not been completely done.

Thirdly, removability. A pulpless tooth is next door neighbor to a crowned root and the ready removal of the filling, to admit the introduction of a pivot is a great convenience. Occasionally, also; it may be necessary to remove the filling to reach an abscess.

A proper combination and manipulation of oxychloride of zinc cement, low heat gutta-percha and electrozone will give a filling which will fulfill the above requirements. First wind two or three fine broaches or old Donaldson bristles with shreds of cotton and lay them on the operating table beside a mixing slab on which is placed two or three low heat gutta-percha cones, one drop of electrozone (concentracted) and the proper proportion of liquid and powder of the cement. Next, having the canal dry, incorporate the cement powder with electrozone and add sufficient of the cement liquid to make a creamy paste, which is immediately picked up by one of the cotton twisted broaches and pumped into the canal, repeating this until there is a slight indication of pain, or until you are confident that the filling has reached the apex. Follow this with one of the gutta-percha cones very slightly warmed on a canal plugger and the work is done. The combination also prevents the cement from setting hard. The pumping process insures the cement reaching the apex and the lining of the sides of the canal to which it will adhere The packing in of the low heat gutta percha compresses the cement, forcing it still more into place in every direction and also leaves a gutta-percha core to facilitate removal should it ever be necessary. The beginning of the canal should be large enough to give the broach piston proper play, while the remainder of the canal need only be enlarged sufficiently to admit the finest Donaldson cleanser.—*Items of Interest.*

